# Mechanisms of action of *Sappan lignum* for prostate cancer treatment: network pharmacology, molecular docking and experimental validation

**DOI:** 10.3389/fphar.2024.1407525

**Published:** 2024-09-10

**Authors:** Wenna Li, Honglin Jiang, Weina Zhang, Qiuyue Sun, Qiaoli Zhang, Jingnan Xu, Jinchang Huang, Yuxiang Wan

**Affiliations:** ^1^ The Third Affiliated Hospital, Beijing University of Chinese Medicine, Beijing, China; ^2^ Institute of Acupuncture and Moxibustion in Cancer Care, Beijing University of Chinese Medicine, Beijing, China

**Keywords:** prostate cancer, *Sappan lignum*, network pharmacology, molecular docking, p53 signaling pathway, experimental validation *in vitro*

## Abstract

**Background:**

Prostate cancer (PCa) is the most common non-cutaneous malignancy in men globally. *Sappan lignum*, which exists in the heartwood of Caesalpinia sappan L., has antitumor effects; however, its exact mechanism of action remains unclear. This study elucidated the underlying mechanisms of *Sappan lignum* in PCa through network pharmacology approaches and molecular docking techniques. Moreover, the therapeutic effects of *Sappan lignum* on PCa were verified through *in vitro* experiments.

**Methods:**

The constituent ingredients of *Sappan lignum* were retrieved from the HERB database. Active plant-derived compounds of *Sappan lignum* were screened based on gastrointestinal absorption and gastric drug properties. Disease targets for PCa were screened using unpaired and paired case datasets from the Gene Expression Omnibus. Intersection targets were used for gene ontology and Kyoto encyclopedia of genes and genomes (KEGG) pathway enrichment analysis. Core targets were identified through topological analysis parameters and their clinical relevance was validated through The Cancer Genome Atlas database. The affinity between the phytochemicals of *Sappan lignum* and core proteins was verified using the molecular docking technique. Validation experiments confirmed the significant potential of *Sappan lignum* in treating PCa.

**Results:**

Twenty-one plant-derived compounds of *Sappan lignum* and 821 differentially expressed genes associated with PCa were collected. Among 32 intersection targets, 8 were screened according to topological parameters. KEGG analysis indicated that the antitumor effects of *Sappan lignum* on PCa were primarily associated with the p53 pathway. The molecular docking technique demonstrated a strong affinity between 3-deoxysappanchalcone (3-DSC) and core proteins, particularly cyclin B1 (CCNB1). CCNB1 expression correlated with clinicopathological features in patients with PCa. Experimental results revealed that 3-DSC exhibited anti-proliferative, anti-migratory, and pro-apoptotic effects on 22RV1 and DU145 cells while also causing G2/M phase cell cycle arrest, potentially through modulating the p53/p21/CDC2/CCNB1 pathway.

**Conclusion:**

This research highlights the promising therapeutic potential of *Sappan lignum* in treating PCa, with a particular focus on targeting the p53 pathway.

## 1 Introduction

Prostate cancer (PCa) is the most prevalent non-cutaneous malignancy and the primary cause of cancer-related deaths in men worldwide (Rizzo et al., 2022). However, delayed diagnosis often leads to higher mortality due to delayed diagnosis (Sekhoacha et al., 2022). PCa causes an estimated 307,000 fatalities and 1.1 million new cases annually (Xue et al., 2022). The rise in incidence is attributed to increased life expectancy and lifestyle changes. In Asia alone, more than 297,000 new cases of PCa are diagnosed each year, representing 23.3% of global cases, with over 118,000 deaths reported, accounting for approximately 33% of all PCa-related deaths (Zhu et al., 2020).

The primary treatment modalities for PCa include radical prostatectomy, endocrine therapy, chemotherapy, radiotherapy, and immunotherapy. Currently, aggressive PCa treatment options have adverse effects on urinary continence and erectile dysfunction (Sebesta and Anderson, 2017). In clinical practice, the majority of patients with advanced PCa develop metastatic castration-resistant PCa and subsequently become resistant to chemotherapy. The resistance poses a significant challenge in terms of management and significantly impacts patients’ quality of life (Rezaeian et al., 2023). Therefore, it is crucial to promptly discover a safe and effective medicine for treating PCa.

Recent studies are exploring alternative therapeutic approaches, including investigating medicinal plants to develop novel anticancer medications. Plant-derived phytochemicals have demonstrated potential as anticancer agents for PCa treatment (Termini et al., 2020). These compounds inhibit various signal transduction pathways in tumor progression and metastasis, enhance anticancer drugs and radiation therapy tolerance, and exhibit multi-targeted antitumor effects (Afrin et al., 2020). Additionally, phytochemicals modulate the tumor microenvironment, inhibit angiogenesis, and have been found to have fewer adverse effects compared to conventional therapies (Kapinova et al., 2019).

Active chemical compounds found in medicinal plants of the subfamily Caesalpinoideae have been reported to exhibit antiproliferative activity by regulating tumor cell apoptosis and oxidative stress (Kamal et al., 2022). *Sappan lignum*, the dry heartwood of Caesalpinia sappan L., is a natural medicinal plant within the subfamily Caesalpiniaceae extensively distributed in subtropical and tropica1 regions worldwide. Studies have reported that *Sappan lignum* possesses antioxidant, antibacterial, anti-inflammatory, vasodilatory, and immunomodulatory properties, as well as antitumor effects (Chen et al., 2021; Wang et al., 2023). Protosappanin B (PSB), a pivotal extract derived from *Sappan lignum,* has been demonstrated to effectively suppress the proliferation and migratory capabilities of colon cancer cells, inducing apoptosis and suggesting its potential as a novel medication to treat tumor (Zheng et al., 2020).

Network pharmacology is a rapidly developing interdisciplinary field that combines biology, pharmacology, mathematics, and computer science to analyze the precise mechanism of drug action by constructing drug components-targets-pathways-diseases network. Network pharmacology utilizes bioinformatics and visualization techniques, including high-throughput screening and multiple network models, to analyze and forecast the mechanism of drug action (Feng et al., 2023). This approach has evolved into a comprehensive tool for investigating the potential active components of drugs and the mechanisms underlying disease prevention and control (Zhen et al., 2023). Recently, the research concepts within network pharmacology have advanced significantly (Zhao et al., 2023). The pharmacological effects and mechanisms of action of these compounds can be thoroughly understood through computational simulation, clinical data mining, and experimental investigations, thereby providing a novel approach to drug development (Khan and Lee, 2023).

Currently, studies have found that *Sappan lignum* has multi-targeted anti-inflammatory and antioxidant effects (Kang et al., 2021; Wang et al., 2023). However, no study has reported the potential mechanisms of *Sappan lignum* in PCa through network pharmacology or molecular docking techniques. Additionally, the anticancer effects of *Sappan lignum* on various PCa cells remain largely unexplored. *In vitro* studies have shown that the active ingredient of *Sappan lignum* inhibits the proliferation of PC3 PCa cells and suppresses cell cycle progression in a concentration-dependent manner (Lv et al., 2022). We believe that the possible signaling pathways and targets of *Sappan lignum* for PCa treatment can be examined through network pharmacology in a holistic, systematic, and comprehensive manner.

**FIGURE 1:**
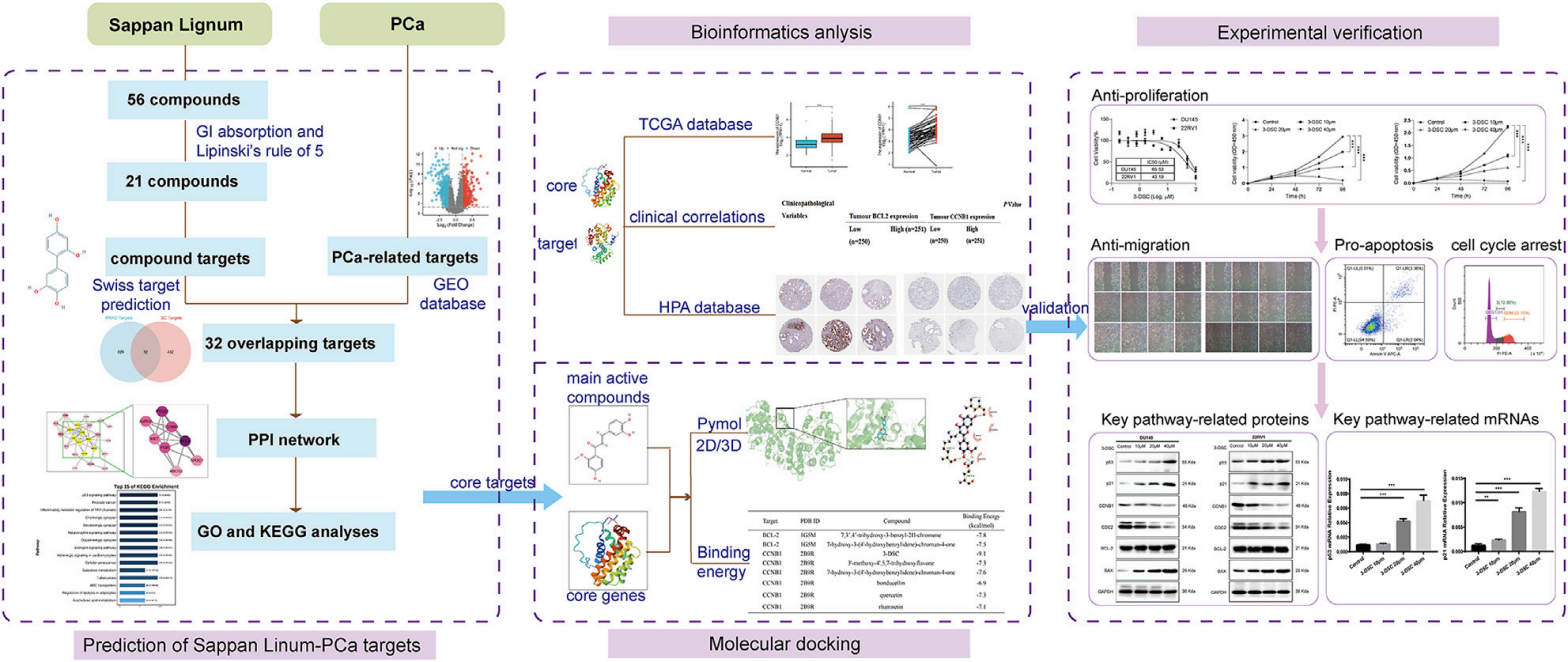
The comprehensive flowchart of this research.

This investigation aimed to determine the active constituents of *Sappan lignum* with anti-PCa effects and construct a network for potential target modulation through network pharmacology. Based on data from the gene expression omnibus (GEO) dataset, we identified potential targets associated with PCa. We developed an “active chemical ingredient-disease target” network utilizing protein-protein interaction data. Subsequently, we screened the core targets and performed pathway enrichment analysis. Furthermore, we visualized the molecular docking interactions between the core components and PCa targets, selecting the core compounds with significant therapeutic effects. We used bioinformatic analysis to validate the target proteins of *Sappan lignum* for PCa treatment. *In vitro* cultures of PCa cells were treated with the active ingredient of *Sappan lignum* to provide a basis for developing new drugs targeting these core active compounds. The workflow is illustrated in Figure 1.

## 2 Materials and methods

### 2.1 *Sappan lignum* compounds screening and target fishing

HERB (http://herb.ac.cn) database (Fang et al., 2021) encompasses Traditional Chinese Medicine Integrated Database (TCMID), SymMap, Traditional Chinese Medicine Information Database (TCM-ID), and Traditional Chinese Medicine Systems Pharmacology (TCMSP) databases for an extensive collection of chemical constituents of *Sappan lignum*. All components’ molecular formulas and smile information were standardized according to PubChem (https://pubchem.ncbi.nlm.nih.gov/) (Kim et al., 2021). The active ingredients and target proteins of *Sappan lignum* were obtained from the HERB database. Gastrointestinal (GI) absorption and Lipinski’s rule of 5 for oral drug-likeness (Lipinski et al., 2001) provided by the SwissADME database (http://www.swissadme.ch/) (Hernandez et al., 2024) were utilized to select the active chemical compounds. The targets of the active constituents were subsequently predicted using SwissTargetPrediction (http://www.swisstargetprediction.ch/) (Zhang et al., 2021).

### 2.2 Retrieval of PCa-associated genes

The GSE46602 and GSE3325 datasets of PCa were retrieved from the GEO database (http://www.ncbi.nlm.nih.gov/geo/) (Clough et al., 2024) of the National Center for Biotechnology Information (NCBI). Before the analysis of differentially expressed genes (DEGs), data quality was assessed through principal component analysis (PCA) (Martins et al., 2019) to ensure that the GSE datasets were usable for differential analysis. Screening criteria for DEGs in PCa and non-cancerous tissues were adjusted to *p* < 0.05 and |log FC| > 1.

### 2.3 Targets gathering and network construction

The targets of *Sappan lignum* and PCa were compiled, deduplicated, and represented in a Venn diagram to illustrate the intersection of targets. After taking the intersection, we obtained 32 targets, the possible therapeutic targets of *Sappan lignum* in the context of PCa treatment. These targets were imported using the String platform (https://www.stringdb.org/) with the species “*Homo sapiens*.” The obtained intersection targets were imported into Cytoscape software (version 3.9.1) to establish the intersection target library and visualize the protein-protein interaction (PPI) network. After screening by the median, eight key targets in the network were found based on the topological degree, closeness centrality, and betweenness centrality parameters, according to our previous research methods (Wan et al., 2019).

### 2.4 GO functional enrichment and KEGG pathway analysis

The biological information database KEGG Orthology-based Annotation System (KOBAS) (http://www.bioinfo.org/kobas) provides exhaustive and systematic functional annotation data for large-scale genes or proteins, primarily for functional and pathway enrichment analysis of differential genes (Wu et al., 2006). To analyze the enrichment of pathways in GO and the KEGG, we utilized the KOBAS platform. The filter criteria were *p* < 0.05. GO annotations and KEGG pathways were visualized using R software (version 4.3.1).

### 2.5 Verification in the PCa cohort

For the two screened targets, differential expression between PCa and non-tumor was analyzed by the Wilcoxon test from the Cancer Genome Atlas (TCGA, https://tcga-data.nci.nih.gov/tcga/) database (Neuberger et al., 2023). Tumor samples in patients with PCa were retrieved from the TCGA database for correlation analysis of clinical data and pathological features. It included Tumor-Node-Metastasis (TNM) classification, residual tumor size, zonal tumor origin, histologic of “Gleason score” evaluation, and serum markers of PCa. We screened for underlying PCa-associated progression with the involvement of core proteins using a chi-square test. The Human Protein Atlas (HPA) database (https://www.proteinatlas.org/) is a comprehensive human gene and protein expression of information resources, integrating proteomics data from multiple tissues and cell types (Digre and Lindskog, 2021)**.** We verified core protein expression to preserve the accuracy of results using the HPA database. Based on the analysis of clinical correlation data, we further observed the immunohistochemical expression of differential key targets in PCa and non-PCa tissues.

### 2.6 Molecular docking of candidate targets and active ingredients

We used the PubChem (https://pubchem.ncbi.nlm.nih.gov/) to import the structures of chemicals that docked to important proteins (Kim et al., 2022), including 7,3′,4′-trihydroxy-3-benzyl-2H-chromene, 7-hydroxy-3-(4′-hydroxybenzylidene)-chroman-4-one, 3-DSC, 3′-methoxy-4′,5,7-trihydroxyflavone, bonducellin, quercetin, and rhamnetin. We minimized the energy with Chem 3D and selected the mol2 format, and then downloaded B-cell lymphoma-2 (BCL-2) and cyclin B1 (CCNB1) protein crystal structures from the RCSB Protein Data Bank (PDB) database (https://www.rcsb.org, accessed on 6 January 2024) (Petros et al., 2001; Petri et al., 2007). We used Pymol software (version 1.5.6) to delete water molecules and small molecules, generated pdb format files of target receptor proteins, imported them into AutoDock Tools software for neutral hydrogenation, charge calculation, and generated pdbqt format files. The treated compounds were used as small molecule ligands, and the protein targets as receptors. We performed molecular docking through AutoDock Tool software (version 1.5.6) and used the Grid function to set the size and positional parameters of the active pockets. The central position of the Grid Box was determined based on the interaction between the small molecules and the targets (BCL-2: center_x = 3.664, y = −4.153, z = 2.012; CCNB1: center_x = −65.873, y = 41.523, z = −11.34). The size of the pockets was set according to the BCL-2 and CCNB1 ligand sizes, which were 40 × 40 × 40 and 60 × 60 × 60 in length, width and height, respectively. Molecular docking was executed utilizing Vina to ascertain the binding energies between the chemical components and the two target proteins. Flexible bonds of small molecule ligands were set to be rotatable, while the receptor proteins were set to semi-flexible docking (Trott and Olson, 2010). The results were evaluated based on the binding energies and binding sites. As the binding energy decreased, the binding activity and affinity also decreased. The docking results were visualized by Pymol software (version 2.3.4).

### 2.7 Experimental verification

#### 2.7.1 Cell culture and treatments

Our study obtained human PCa cell lines 22RV1 and DU145 from the Cell Resource Center, Institute of Basic Medical Sciences. 22RV1 and DU145 cells were cultured in RPMI 1640 and DMEM medium (Gibco Carlsbad) + 10% fetal bovine serum (FBS) + 1% penicillin-streptomycin and both were cultured at 37°C in a 5% CO_2_ incubator (Cheng et al., 2021). Cells were passaged at a confluency of approximately 80% fusion, and the logarithmic growth phase was used to extract cells for experimental purposes after three passages. DU145 and 22RV1 cells were treated with 3-DSC for antitumor purposes, obtained from MedChemExpress.

#### 2.7.2 CCK-8 assay

The cells were blown into single-cell suspensions and inoculated into 96-well plates with 100 μL per well. For cytotoxicity assays, the cell density was approximately 20%–25%. The culture was incubated for 24 h at 37°C in a 5% CO_2_ incubator. Different concentrations of drug-containing media depended on the requirements of drug concentration. After 24 h of treatment, 100 μL medium containing 10% CCK-8 (CK04, Dojindo) was added into each well and incubated in the cell incubator for 1–2 h. The absorbance was measured at 450 nm.

For proliferation assay, cells were seeded at a 5%–7% density and placed in the 37°C in a 5% CO_2_ incubator for 24 h. Experimental groups were treated with various doses of 3-DSC (10, 20, and 40 μM) for 24, 48, and 72 h, while the control group (0 μM) was treated with RPMI 1640 containing 10% FBS. Subsequently, CCK-8 reagent was added to cells, and the absorbance was measured.

#### 2.7.3 Cell scratch test

Cells were put into six-well plates with 2 mL of medium in each well and cultured in an incubator for 24 h. Experimental and control groups were exposed to different concentrations of DSC and RPMI 1640. We used 200 µL tips to create a consistent width cell scratch in the center of the six-well plate after 24 h or 48 h and rinsed with PBS to remove cellular debris produced by scratching. Then a complete medium was added, and photographs were recorded as 0 h. By comparing the photographs at various time points, the migration and growth of cells near scratches could be observed.

#### 2.7.4 Cell apoptosis detection

2 mL of single-cell suspension per well was inoculated onto a 6-well plate with a cell density of 20%. After treatment with 3-DSC or medium for 24 h, we collected 5 × 10^4^ cells, added Annexin V-FITC and propidium iodide (PI) to incubate at room temperature and dark for 20 min, then placed them in an ice bath and performed flow cytometry detection using CytoFLEX S flow cytometer (Beckman Coulter Life Sciences). PI emits red fluorescence, whereas Annexin V-FITC emits green fluorescence.

#### 2.7.5 Cell cycle distribution detection

Cells were digested into single-cell suspensions by trypsin, fixed with 70% ethanol at 4°C for 30 min, and precipitated by centrifugation. Excess RNA was removed by adding RNase A. Each tube of cell samples was added PI staining solution and incubated in a warm bath at 37°C temperature and light avoidance for 30 min. We utilized flow cytometry to analyze the distribution of the cell cycle by detecting red fluorescence at the excitation wavelength of 488 nm.

#### 2.7.6 Western blotting analysis

We collected two types of cells, added RIPA lysis buffer (KeyGEN BioTECH) and centrifugated to obtain the whole cell protein extraction solution. The protein concentrations of cells were estimated using the BCA Protein Assay Kit (KeyGEN BioTECH). A protein sampling buffer was added for denaturation. We prepared 10% separation glue, and separated proteins were electro-transferred onto polyvinylidene fluoride (PVDF) membranes at constant current and low temperature. Ponceau stain was used to verify the membrane transfer efficiency. The blots were obstructed using a 5% solution of non-fat dry milk at 37°C on a shaking platform for 1 h. Then, we dilute d the primary antibody to the optimal concentration using l×TBST, including anti-GAPDH (1:2500, ab9485, Abcam), anti-p53 (1:1000, ab32049, Abcam), anti-p21 (1:5000, ab109520, Abcam), anti-Cyclin B1 (1:50000, ab32053, Abcam), anti-CDK1 (1:10000, ab133327, Abcam, United Kingdom), anti-Bcl2(1:1000, ab182858, Abcam) and anti-Bcl2(1:10000, ab32503, Abcam). The membranes with the proteins were incubated at overnight 4°C, then the secondary antibody HRP-conjugated Affinipure Goat Anti-Rabbit IgG (1:2000, SA00001-2, Proteintech) was added and appropriate amounts of enhanced chemiluminescence (KGC4601-100, KeyGEN BioTECH) were dropwise added near the location of target proteins. Finally, protein bands were exposed by a gel imaging system and photographed for storage.

#### 2.7.7 Quantitative real-time PCR (qRT-PCR)

Cellular total RNA was isolated utilizing TRIzol and chloroform (Sinopharm Chemical Reagent) precipitated by adding isopropanol and resuspended. Sequences of qPCR primers are listed in Table 1. cDNA synthesis was performed by GoScript™ Reverse Transcription System (Promega). AceQ qPCR SYBR Green Master Mix (Vazyme) was used for the amplification reaction system. The relative expression levels of BCL2 associated X protein (BAX), BCL-2, CCNB1, cell division cycle 2 (CDC2), p53, and p21 mRNA were measured by qRT-PCR.

**TABLE 1:** Primer sequence information in this study.

Gene	Species	Primer sequence
p53	Human	F: 5′-GCT​GCT​CAG​ATA​GCG​ATG​GTC​T-3′
	Human	R: 5′-CAA​CCT​CAG​GCG​GCT​CAT​AG-3′
p21	Human	F: 5′-ACC​ACT​GGA​GGG​TGA​CTT​C-3′
	Human	R: 5′-CGG​CGT​TTG​GAG​TGG​TAG-3′
CCNB1	Human	F: 5′-GCC​TAT​TTT​GGT​TGA​TAC​TGC​CTC-3′
	Human	R: 5′-CTC​CAT​CTT​CTG​CAT​CCA​CAT​C-3′
CDC2	Human	F: 5′-AAA​CTA​CAG​GTC​AAG​TGG​TAG​CC-3′
	Human	R: 5′-TCC​TGC​ATA​AGC​ACA​TCC​TGA-3′
BCL-2	Human	F: 5′-ATC​GCC​CTG​TGG​ATG​ACT​GA-3′
	Human	R: 5′-GAG​ACA​GCC​AGG​AGA​AAT​CAA​AC-3′
BAX	Human	F: 5′-TTT​TGC​TTC​AGG​GTT​TCA​TCC​A-3′
	Human	R: 5′-TGC​CAC​TCG​GAA​AAA​GAC​CTC-3′

“F” represents “forward sequence; ” “R” represents “reverse sequence.”

#### 2.7.8 Statistical analysis

Experimental data was expressed as mean ± standard deviation (SD). To determine differences between the treatment and control groups, the *t*-test or Mann-Whitney *U* test was utilized. *p*-value < 0.05 was deemed to indicate statistical significance. All of the statistical analyses listed above were conducted utilizing SPSS software (Version 20.0, IBM, Chicago, IL, United States).

## 3 Results

### 3.1 *Sappan lignum* compounds screening and target fishing

**FIGURE 2:**
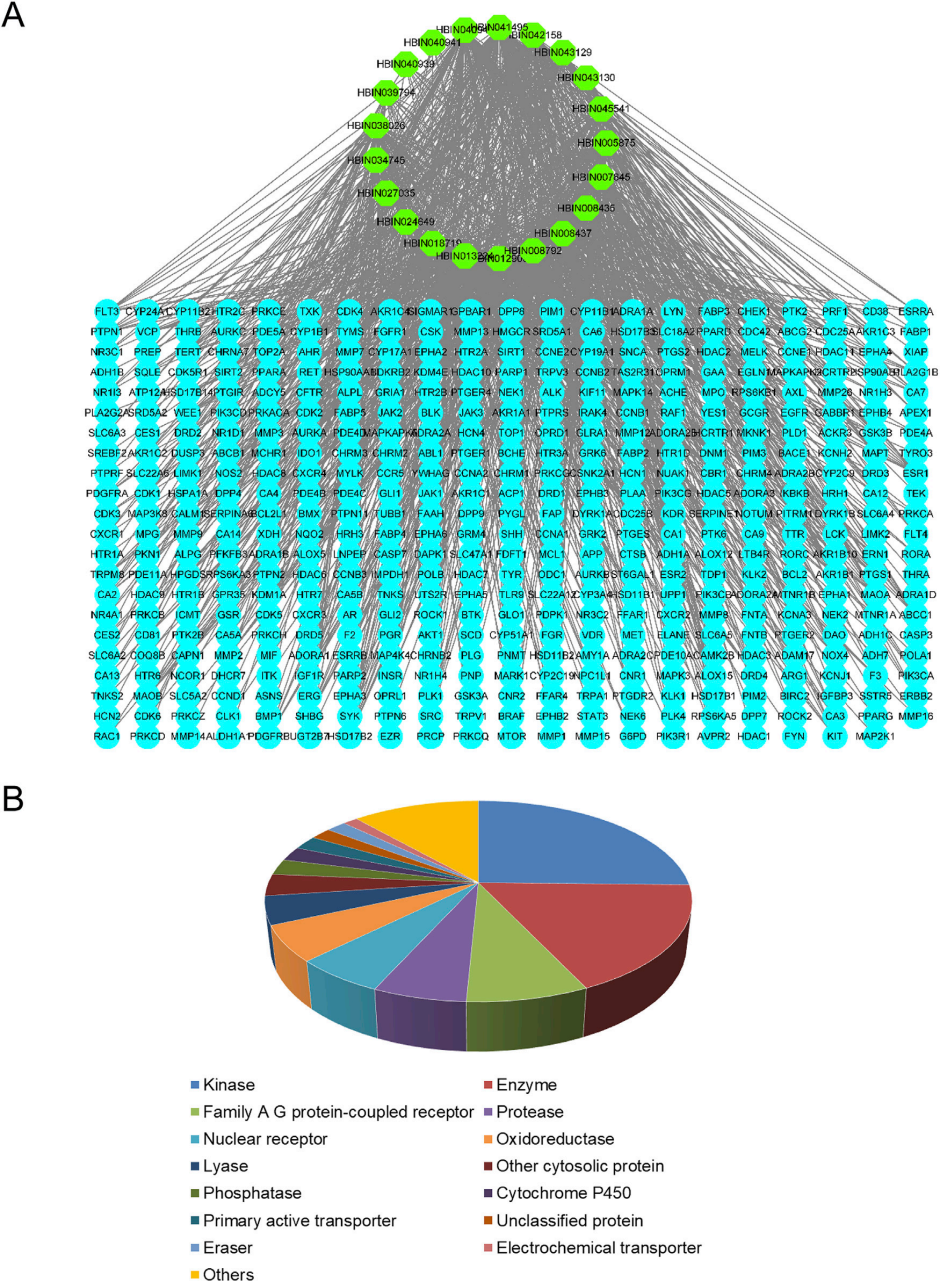
Active ingredient-targets of *Sappan lignum*. **(A)** Active ingredient-targets network. The green ellipse represents the 21 active components of *Sappan lignum*, and the blue ellipse signifies the corresponding target of the components. The edges represent the interaction between active compounds and targets **(B)** Key target types of active ingredients. Different colors represent different attribute classification targets of active ingredients, involving a total of 14 categories.

Of the 56 chemical ingredients of *Sappan lignum* obtained from the HERB database, 21 compounds were screened through SwissADME. Detailed information, GI absorption, and Lipinski’s rule of 5 are given in Table 2. Lipinski’s rule of 5, which evaluates oral drug-likeness in pharmacokinetic analysis, combines criteria from the Lipinski, Ghose, Veber, Egan, and Muegge rules. According to the screening criteria of “High” GI and 5 “yes” results of oral drug-likeness, 21 bioactive components of *Sappan lignum* were selected. Based on the chemical structure, 471 possible targets were predicted through target fishing on the Swiss target prediction platform. Cytoscape 3.9.2 was used to construct the component-targets network (Figure 2A). In total, 32 target classes were involved in related targets (Figure 2B). The top 10 target types were kinase (25.34%), enzyme (17.64%), G protein-coupled receptor (7.77%), protease (5.98%), nuclear receptor (5.83%), oxidoreductase (5.75%), lyase (4.56%), other cytosolic protein (3.51%), phosphatase (2.54%), and cytochrome P450 (2.32%) (Figure 2B).

**TABLE 2:** 21 active compounds of *Sappan lignum* and their GI and Lipinski’s rule of 5.

Compound name	PubChem CID	Molecular Formulas	GI	Lipinski’s rule of 5	Structure
2′-methoxy-3,4,4′-trihydroxychalcone	5319493	C_16_H_14_O_5_	high	5 Yes	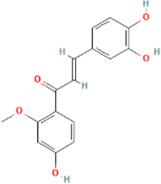
3,9-dihydroxy-8-methoxydibenzo [b, d] pyran-6-one	102178085	C_14_H_10_O_5_	high	5 Yes	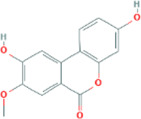
3-deoxysappanchalcone	13846660	C_16_H_16_O_5_	high	5 Yes	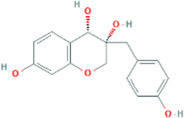
3-deoxysappanone b	91825567	C_16_H_14_O_5_	high	5 Yes	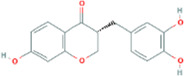
3′-methoxy-4′,5,7-trihydroxyflavone	5280666	C_16_H_12_O_6_	high	5 Yes	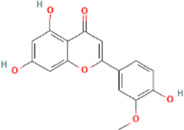
7,3′,4′-trihydroxy-3-benzyl-2H-chromene	71307370	C_16_H_14_O_4_	high	5 Yes	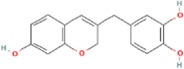
7-hydroxy-3-(4′-hydroxybenzylidene)-chroman-4-one	91980449	C_16_H_12_O_4_	high	5 Yes	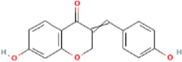
bonducellin	14079439	C_17_H_14_O_4_	high	5 Yes	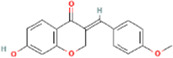
(E)-2-nonenal	5283335	C_9_H_16_O	high	5 Yes	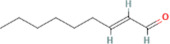
gallic acid-3-O-(6′-O-galloyl)-glucoside	11972353	C_20_H_20_O_14_	high	5 Yes	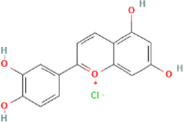
menthol	1254	C_10_H_20_O	high	5 Yes	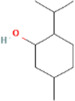
oleic acid	445639	C_18_H_34_O_2_	high	5 Yes	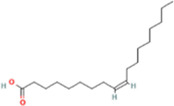
phytol	5366244	C_20_H_40_O	high	5 Yes	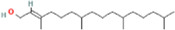
protosappanin a	128001	C_15_H_12_O_5_	high	5 Yes	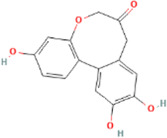
protosappanin a dimethyl acetal	11,347,693	C_17_H_18_O_6_	high	5 Yes	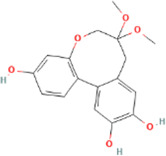
protostemonine	25256772	C_23_H_31_NO_6_	high	5 Yes	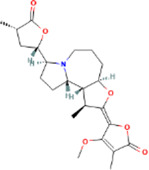
quercetin	5280343	C_15_H_10_O_7_	high	5 Yes	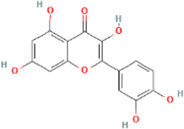
rhamnetin	5281691	C_16_H_12_O_7_	high	5 Yes	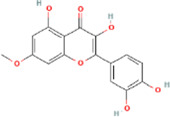
sappanchalcone	5319493	C_16_H_14_O_5_	high	5 Yes	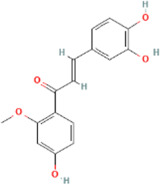
sappanin	5321124	C_12_H_10_O_4_	high	5 Yes	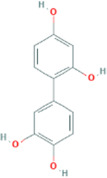
Taraxerol	92097	C_30_H_50_O	high	5 Yes	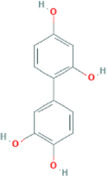

### 3.2 Investigation of PCa-related targets

**FIGURE 3:**
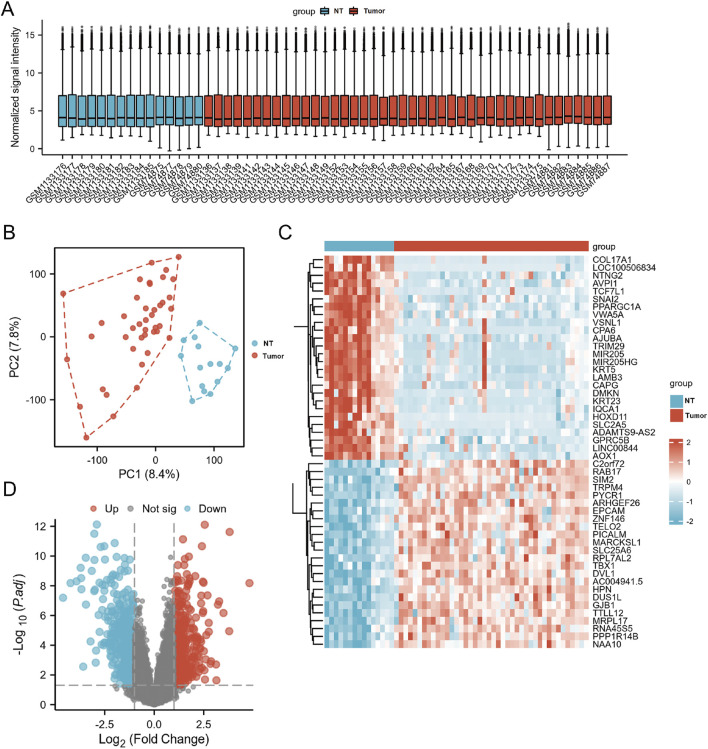
The DEGs in PCa and control samples were analyzed using data from GSE46602 and GSE3325. **(A)** Box plot of normalized analysis. The GSE46602 and GSE3325 data were combined for standardization analysis, with the number of sample datasets on the *x*-axis and the normalized signal intensity on the *y*-axis. **(B)** PCA of GSE46602 and GSE3325. The red dots represent tumor tissue, and the blue dots represent normal tissue. **(C)** Heatmaps of DEGs. Cluster analysis divided DEGs into two groups. The blue and red hues indicate downregulated and upregulated genes, respectively, and darker colors represent more marked differences in expression. **(D)** The volcano diagram of DEGs. Blue dots represent downregulated genes in PCa, grey dots represent genes with no significant difference, and red dots represent upregulated genes.

From the two PCa public datasets, GSE46602 and GSE3325, 15 non-cancer and 42 PCa samples were obtained. Quality-controlled box plot analysis exhibited that the median values of all samples were nearly identical (Figure 3A), indicating a high level of normalization and suitability for further differential gene analysis. Additionally, plot analysis (Figure 3B) exhibited a distinct separation between the normal prostate and PCa groups, suggesting a clear differentiation between the two sample sets. The parameters were modified to find 821 differentially expressed genes (*p* < 0.05 and |log FC| > 1). A total of 335 upregulated genes and 486 downregulated genes were identified in the study. These findings were visually represented in the volcano plot and hierarchical clustering heat map (Figures 3C,D).

### 3.3 Targets gathering and PPI network construction

**FIGURE 4:**
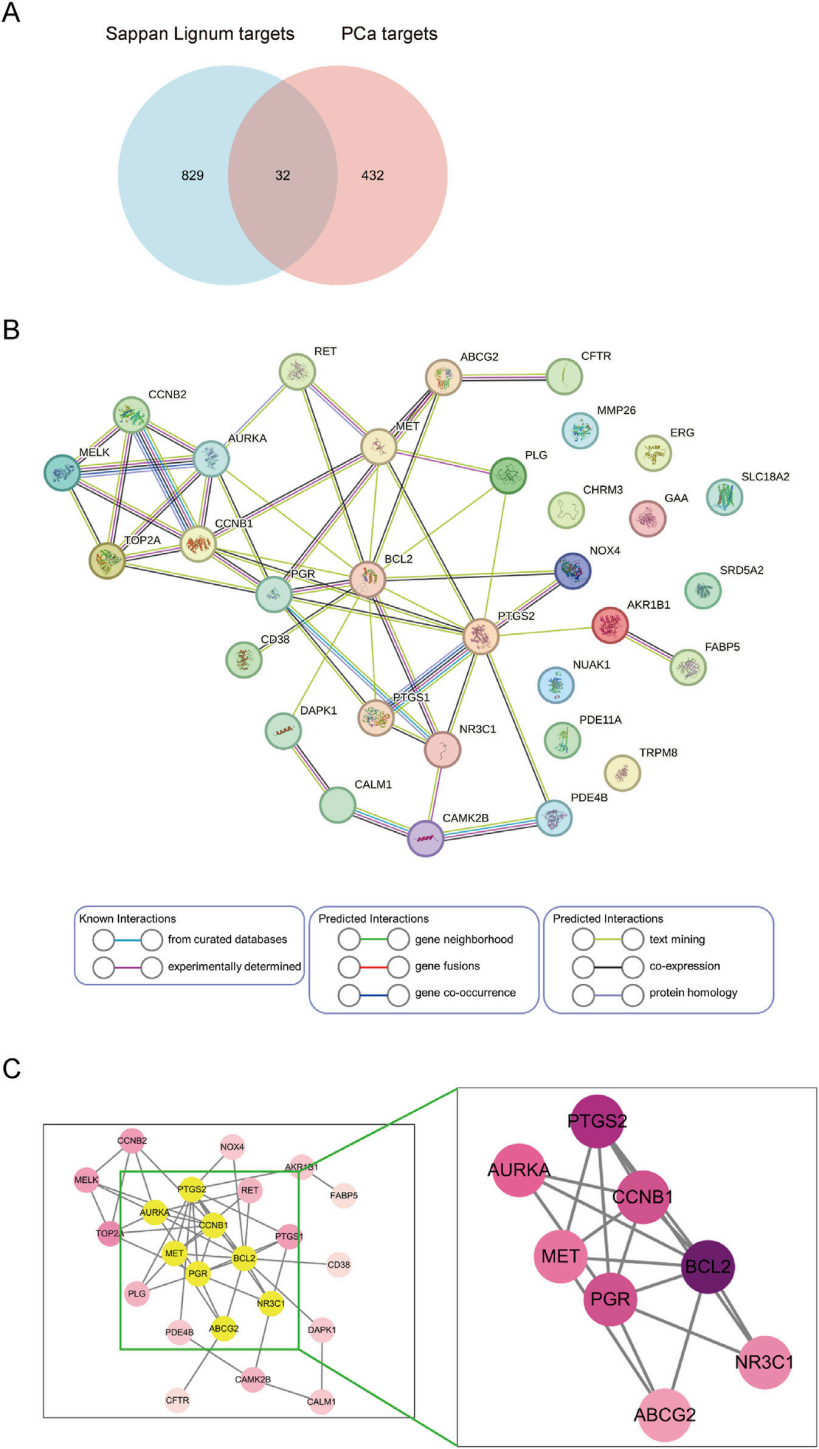
Screening and construction of the PPI network. **(A)** Venn diagram of *Sappan lignum* against PCa has 32 common targets. **(B)** PPI network of 32 overlapping targets. Circles represent therapeutic targets of *Sappan lignum*. The edges indicate the relationship between them, with thicker lines indicating stronger connections. **(C)** Core targets were screened based on the median value of degree, betweenness, and closeness.

We sorted out the relevant targets of *Sappan lignum* (432) and PCa (829) and removed duplicates. Venn established an intersection target library, and the resulting intersection targets were screened out (Figure 4A). The PPI was constructed from 32 intersection targets with 32 nodes and 50 edges (Figure 4B). According to the topological degree, closeness centrality and betweenness centrality parameters, eight core targets in the network were identified after screening by median: BCL-2 (Degree = 13), PTGS2 (Degree = 10), CCNB1 (Degree = 8), PGR (Degree = 8), AURKA (Degree = 7), MET (Degree = 6), NR3C1 (Degree = 5), ABCG2 (Degree = 4) (Figure 4C).

### 3.4 GO functional enrichment and KEGG pathway analysis

**FIGURE 5:**
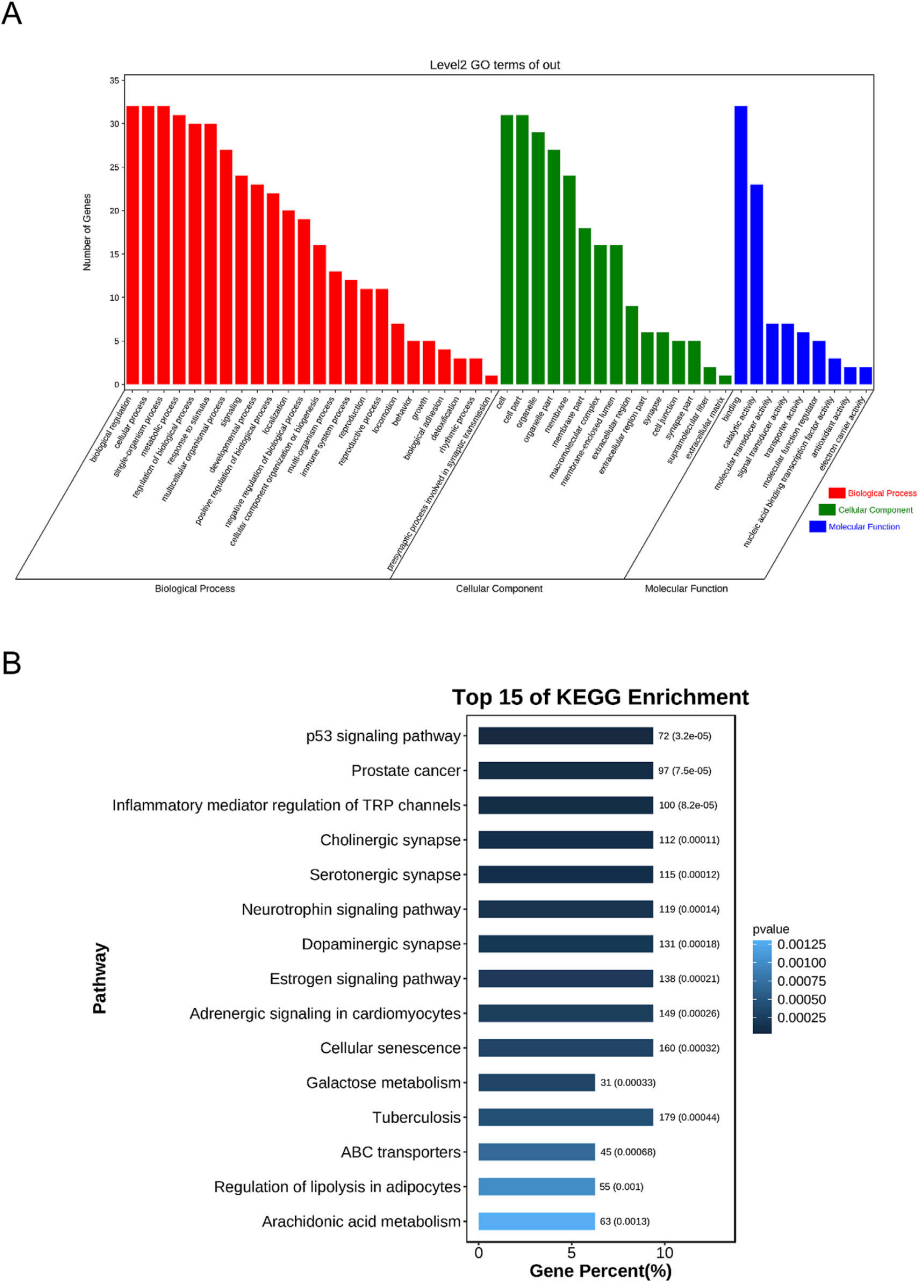
The GO and KEGG enrichment analyses of *Sappan lignum* and PCa genes. **(A)** Overlapped GO terms of *Sappan lignum* and PCa and enrichment analysis. Red, green, and blue represent different GO enrichment analysis pathways: BP, CC, and MF. **(B)** The top 15 pathways of *Sappan lignum* in treating PCa through KEGG enrichment analysis. The *x* and *y*-axes reflect the gene percentage and related pathway names, respectively, with varying shades of color indicating the extent of variation.

To determine the possible therapeutic targets of *Sappan lignum* concerning gene function and signaling pathways, 32 intersection targets of *Sappan lignum* against PCa were entered into the KOBAS platform for KEGG and GO function analysis. Enrichment items for molecular function (MF), biological process (BP), and cellular component (CC) were visualized (Figure 5A). According to the GO enrichment analysis, multiple genes collectively exerted biological effects. *Sappan lignum* primarily interfered with BP involving biological regulation, cellular process, single-organism process, CC comprising a cell, cell part, organelle, and MF, including binding, catalytic activity, and transducer activity in PCa. According to these findings, the anti-PCa effects of *Sappan lignum* can be ascribed to cellular biological regulation.

We performed KEGG pathway analysis to screen the signaling pathways enriched in the 32 intersecting targets (Figure 5B). The top 10 pathways were tumor-associated p53 signaling pathway, PCa, inflammatory mediator regulation of TRP channels, cholinergic synapse, serotonergic synapse, neurotrophin signaling pathway, dopaminergic synapse, estrogenic synapse, adrenergic signaling in cardiomyocytes, and cellular senescence. The above pathways were primarily implicated in PCa disease, p53, cellular senescence, and neural signaling. Utilizing literature research and enrichment analysis, we focused on the p53 signaling pathway, which exhibited the most significant difference among the key targets of *Sappan lignum* for PCa treatment, with the associated proteins BCL-2 and CCNB1.

### 3.5 Verification in the PCa clinical cohort

**FIGURE 6:**
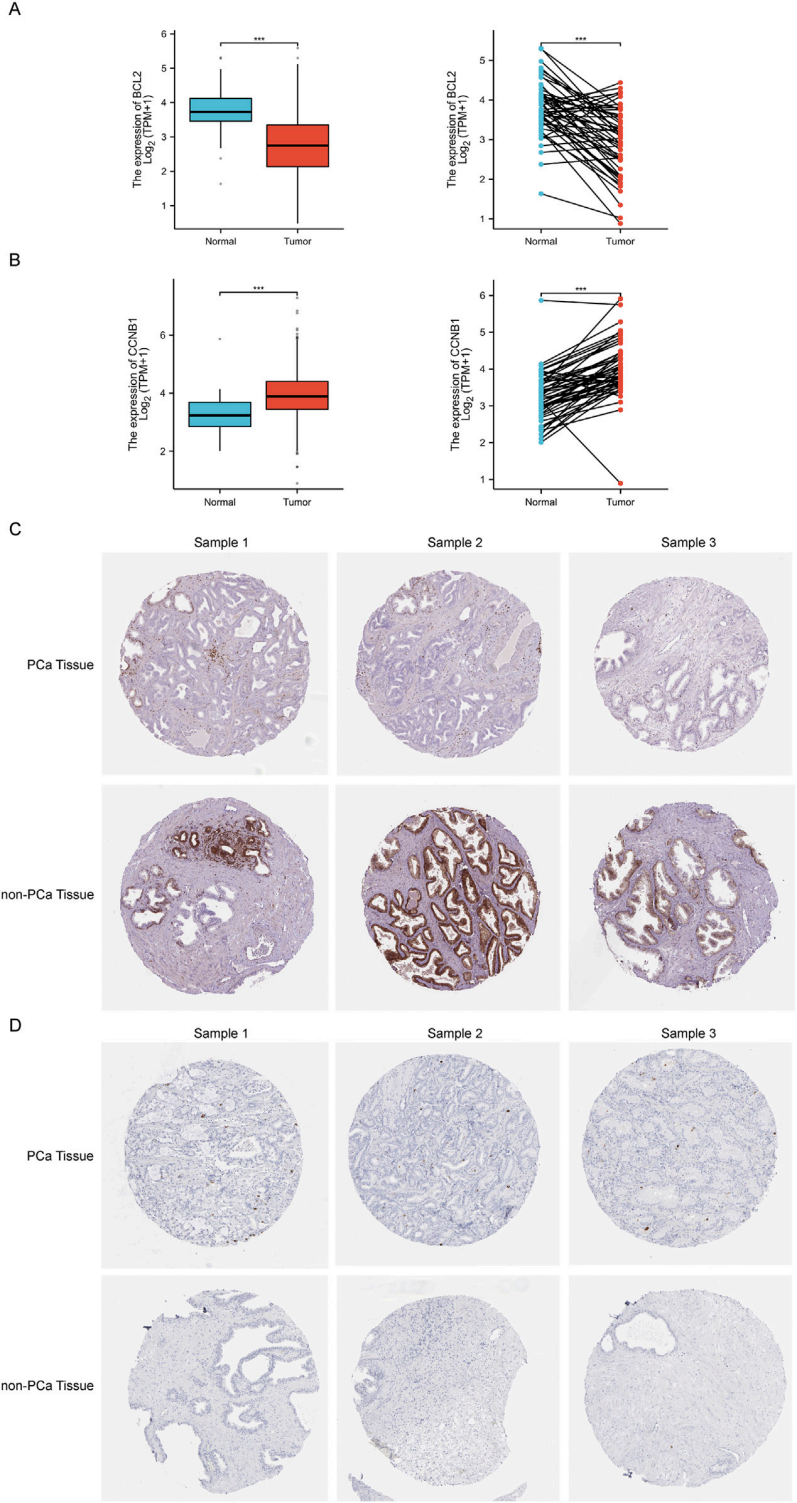
The relative expression levels of core targets BCL-2 and CCNB1 in TCGA and HPA databases. **(A, B)** The left and right figure illustrates the relative expression of BCL-2 and CCNB1 between independent and paired samples of PCa and non-tumor tissues severally. Red for tumor samples, blue for non-tumor samples. ***p < 0.001 compared with PCa group. **(C, D**) Representative immunohistochemical images of BCL-2 and CCNB1 in PCa and normal prostate tissue from the HPA database.

Based on enrichment analysis, we focused on the critical links of *Sappan lignum* in treating PCa on the p53 signaling pathway, which exhibited the most significant differences. For the two potential targets associated with this pathway, we further verified the expression of CCNB1 and BCL-2 in the tissues of PCa patients through TCGA public clinical sample data. Significant expression differences of CCNB1 and BCL-2 were observed in 501 PCa tissue samples compared to 52 non-tumor tissue samples, with PCa tissues exhibiting significantly higher expression of CCNB1 and lower expression of BCL-2. Moreover, 53 paired samples of PCa and adjacent tissues further confirmed the above expression differences (Figures 6A,B). These findings were consistent with the expression trends in the GSE46602 and GSE3325 microarray datasets.

Immunohistochemical detection of key protein expression was studied in the HPA database (Figures 6C,D), which indicated that the expression of BCL-2 was significantly reduced in PCa tissues relative to non-tumorous tissues, while CCNB1 expression was significantly elevated. This is in accordance with the results of the TCGA clinical data.

The PCa clinical cohort was categorized into high and low expression based on the median CCNB1 and BCL-2 expression levels. Correlation analysis of clinical features was then performed (Table 3). The results of our study exhibited a significant association between the expression of CCNB1 and age, residual tumor status, T stage, N stage, and Gleason score. Specifically, upregulation of CCNB1 expression was significantly higher in patients over 60 years, with R0 residual tumor status, T3 stage, N0 stage, and a Gleason score of 7. In conclusion, combined with the clinical cohort information and pathological data of TCGA and tissue and pathology results of the HPA database, we believed that the expression changes of BCL-2 and CCNB1 in the anti-PCA process of *Sappan lignum* may have improved stability.

**TABLE 3:** Relationship between BCL2 and CCNB1 protein expression and clinical characteristic in patients with PCa.

Clinicopathological Variables	Tumour BCL2 expression		*p*-value	Tumour CCNB1 expression		*p*-value
	Low (n = 250)	High (n = 251)		Low (n = 250)	High (n = 251)	
Age, n (%)			0.625			0.005
>60	135 (26.9%)	141 (28.1%)		122 (24.4%)	154 (30.7%)	
≤60	115 (23.0%)	110 (22.0%)		128 (25.5%)	97 (19.4%)	
Residual tumor, n (%)			0.391			0.008
R0 R1 R2	156 (33.2%) 73 (15.5%) 4 (0.9%)	160 (34%) 76 (16.2%) 1 (0.2%)		173 (36.8%) 61 (13%) 1 (0.2%)	143 (30.4%) 88 (18.7%) 4 (0.9%)	
Zone of origin, n (%)			0.633			0.264
Central Multiple	2 (0.7%) 68 (24.5%)	2 (0.7%) 59 (21.3%)		2 (0.7%) 50 (18.1%)	2 (0.7%) 77 (27.8%)	
Peripheral	71 (25.6%)	67 (24.2%)		71 (25.6%)	67 (24.2%)	
Transition	6 (2.2%)	2 (0.7%)		4 (1.4%)	4 (1.4%)	
T stage, n (%)			0.165			<0.001
T2	104 (21.1%)	85 (17.2%)		117 (23.7%)	72 (14.6%)	
T3	139 (28.1%)	155 (31.4%)		127 (25.7%)	167 (33.8%)	
T4	4 (0.8%)	7 (1.4%)		3 (0.6%)	8 (1.6%)	
N stage, n (%)			0.305			0.010
N0	170 (39.7%)	178 (41.6%)		177 (41.4%)	171 (40%)	
N1	34 (7.9%)	46 (10.7%)		28 (6.5%)	52 (12.1%)	
M stage, n (%)			0.982			1.000
M0	232 (50.4%)	225 (48.9%)		227 (49.3%)	230 (50%)	
M1	1 (0.2%)	2 (0.4%)		1 (0.2%)	2 (0.4%)	
PSA (ng/mL), n (%)			0.749			0.050
<4	203 (45.7%)	214 (48.2%)		220 (49.5%)	197 (44.4%)	
≥4	14 (3.2%)	13 (2.9%)		9 (2%)	18 (4.1%)	
Gleason score,n (%)			0.130			<0.001
6	27 (5.4%)	19 (3.8%)		36 (7.2%)	10 (2%)	
7	124 (24.8%)	124 (24.8%)		137 (27.3%)	111 (22.2%)	
8	30 (6.0%)	35 (7.0%)		34 (6.8%)	31 (6.2%)	
9	68 (13.6%)	70 (14%)		42 (8.4%)	96 (19.2%)	
10	1 (0.2%)	3 (0.8%)		1 (0.2%)	3 (0.6%)	
OS event, n (%)			0.745			1.000
Alive	244 (48.7%)	247 (49.3%)		245 (48.9%)	246 (49.1%)	
Dead	6 (1.2%)	4 (0.8%)		5 (1%)	5 (1%)	
DSS event, n (%)			0.371			1.000
No	246 (49.3%)	248 (49.7%)		247 (49.5%)	247 (49.5%)	
Yes	4 (0.8%)	1 (0.2%)		3 (0.6%)	2 (0.4%)	
PFI event, n (%)			0.349			0.070
No	199 (39.7%)	208 (41.5%)		211 (42.1%)	196 (39.1%)	
Yes	51 (10.2%)	43 (8.6%)		39 (7.8%)	55 (11%)	

### 3.6 Molecular docking of candidate targets and related ingredients

**FIGURE 7:**
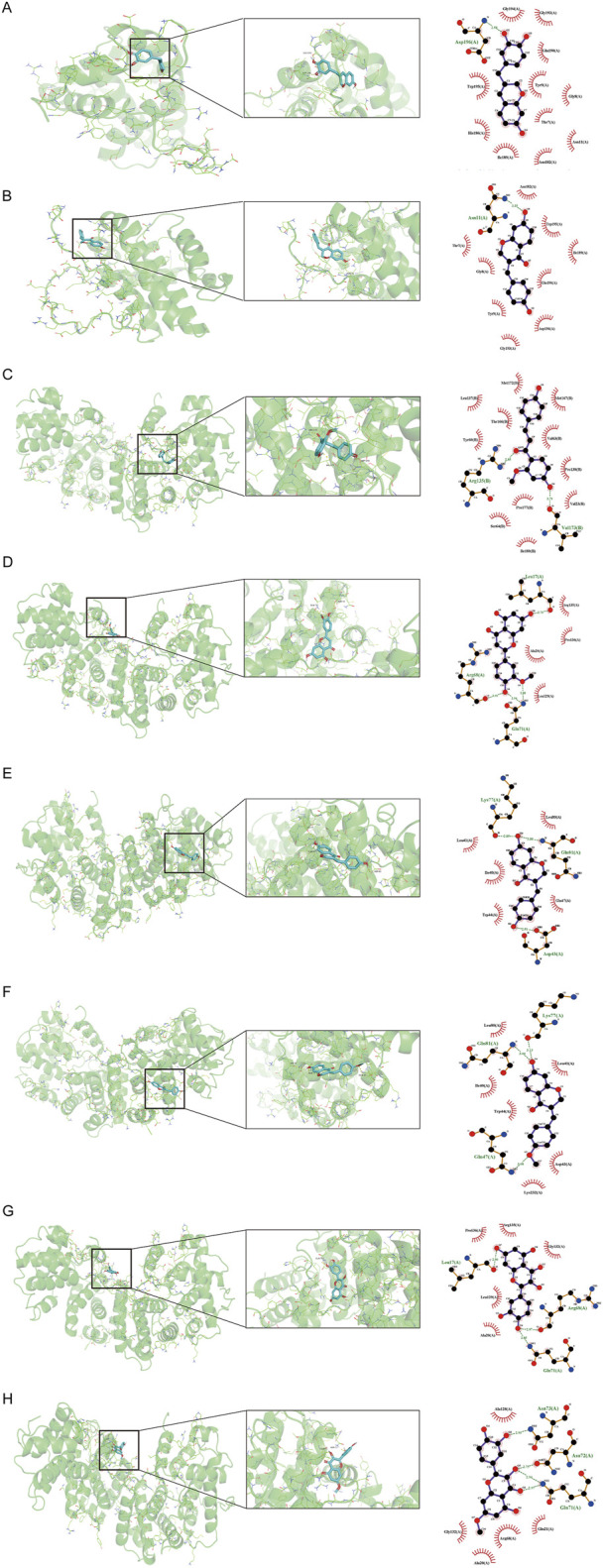
Molecular docking interaction diagram of two hub targets with related compounds. The 3D and 2D binding conformation are given on the left and right. In the 3D interaction, the yellow connection between the compound and the amino acid residue represents hydrogen bonding. The green connection in the 2D interaction represents hydrogen bonding. **(A)** BCL-2 and 7,3′,4′-trihydroxy-3-benzyl-2H-chromene, binding energy = −7.8 kcal/mol. **(B)** BCL-2 and 7-hydroxy-3-(4′-hydroxybenzylidene)-chroman-4-one, binding energy = −7.5 kcal/mol. **(C)** CCNB1 and 3-DSC, binding energy = −9.1 kcal/mol. **(D)** CCNB1 and 3′-methoxy-4′,5,7-trihydroxyflavone, binding energy = −7.3 kcal/mol. **(E)** CCNB1 and 7-hydroxy-3-(4′-hydroxybenzylidene)-chroman-4-one, binding energy = −7.6 kcal/mol. **(F)** CCNB1 and bonducellin, binding energy = −6.9 kcal/mol. **(G)** CCNB1 and quercetin, binding energy = −7.3 kcal/mol **(H)** CCNB1 and quercetin, binding energy = −7.1 kcal/mol.

Based on the identification of top target proteins in the PPI network and validation in the PCa cohort, our study focused on CCNB1 and BCL-2. We analyzed the interactions between key targets and their primary active constituents using molecular docking technology. Docking activity is indicated by a binding energy below 0 kcal/mol, and good docking activity is indicated by a binding energy below −6 kcal/mol. Our findings revealed that the binding energy between compound 3-DSC and CCNB1 was −9.1 kcal/mol, the lowest docking score among all 3-DSC active components and target proteins, suggesting that the binding conformation between them was the most stable. 3-DSC is the best candidate *for Sappan lignum* against PCa; the results are presented in Table 4. PyMOL and LigPlus were employed to analyze the specific docking patterns of active chemical components of *Sappan lignum* with key proteins in the p53 pathway, allowing for the visualization of multiple interactions. Representative images of the 3D and 2D binding conformations are illustrated in Figure 7. BCL-2 interacted with 7,3′,4′-trihydroxy-3-benzyl-2H-chromene, and 7-hydroxy-3-(4′-hydroxybenzylidene)- chroman-4-one and formed a hydrogen bond, respectively. CCNB1 exhibited more hydrogen bonds with the active chemical constituents of *Sappan lignum* than BCL-2. These results indicated that CCNB1 is a crucial target protein in the p53 pathway for the relevant active components of *Sappan lignum*.

**TABLE 4:** Binding energy of target protein docking.

Target	PDB ID	Compound	Binding energy (kcal/mol)
BCL-2	1G5M	7,3’,4’-trihydroxy-3-benzyl-2H-chromene	−7.8
BCL-2	1G5M	7-hydroxy-3-(4′-hydroxybenzylidene)-chroman-4-one	−7.5
CCNB1	2B9R	3-DSC	−9.1
CCNB1	2B9R	3′-methoxy-4′,5,7-trihydroxyflavone	−7.3
CCNB1	2B9R	7-hydroxy-3-(4′-hydroxybenzylidene)-chroman-4-one	−7.6
CCNB1	2B9R	bonducellin	−6.9
CCNB1	2B9R	quercetin	−7.3
CCNB1	2B9R	rhamnetin	−7.1

### 3.7 Experimental verification

#### 3.7.1 3-DSC suppressed cell viability and proliferation of DU145 and 22RV1

**FIGURE 8:**
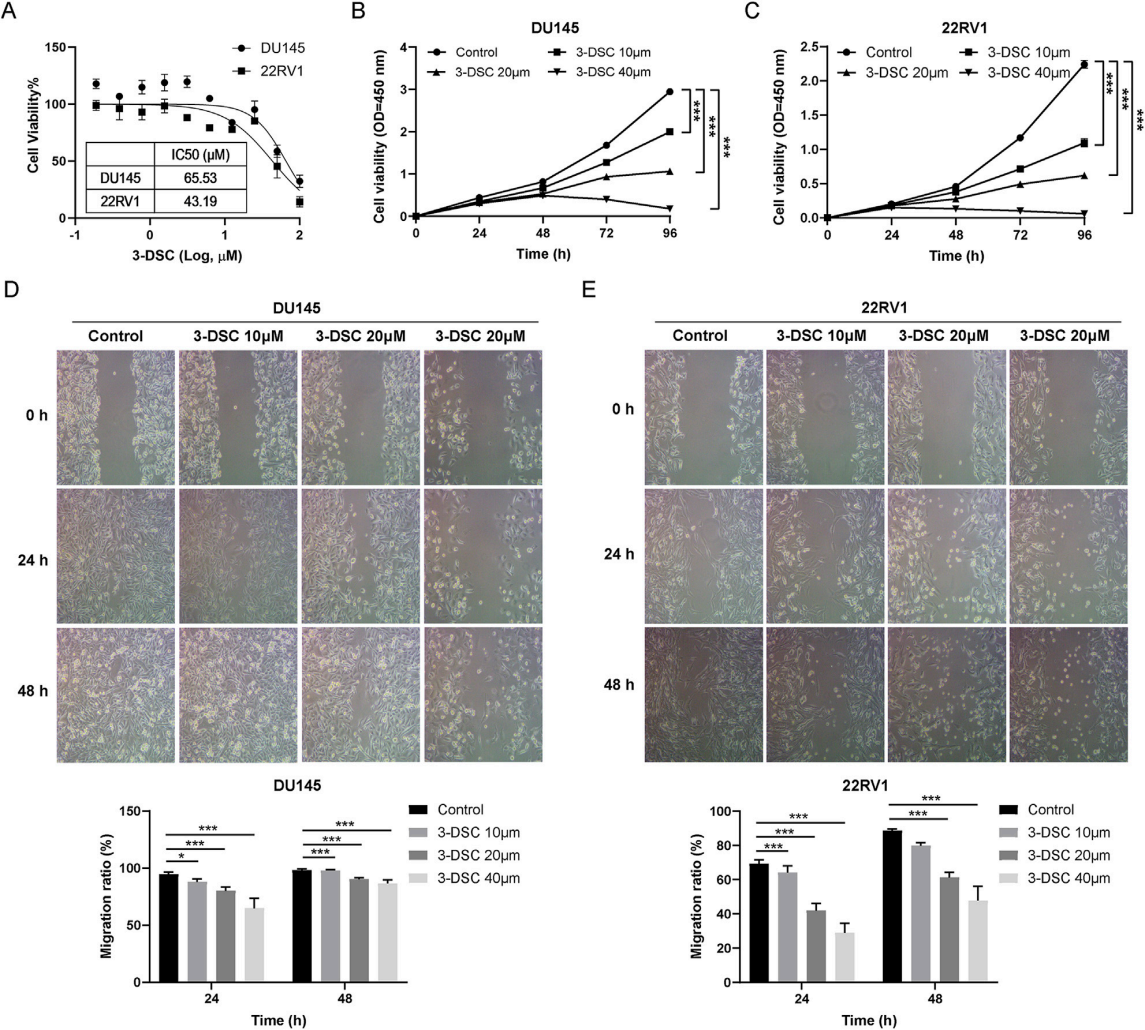
3-DSC restrained the proliferation and migration of 22RV1 and DU145. **(A)** Cell viability of 3-DSC concentration gradient treatment was determined after 24 h by CCK-8 assay. Proliferation of DU145 **(B)** and 22RV1 **(C)** 3-DSC-treated cells were 10 M, 20 M, and 40 M in concentration. Migration of DU145 **(D)** and 22RV1 **(E)** cells after 24 or 48 h 3-DSC treatment. Histograms demonstrate the cell migration ratio for different groups in PCa. Compared to the control group, *p < 0.05, ***p < 0.001.

To determine the cytotoxic impact of 3-DSC on 22RV1 and DU145 cells, the cells were exposed to varying concentrations of 3-DSC for 24 h. At various concentrations, 3-DSC inhibited the proliferation of PCa cells (Figure 8A). The 24 h IC_50_ of 3-DSC for 22RV1 and DU145 cells was 65.53 and 43.19 μM, respectively. The IC_50_ value is an important reference for establishing the gradient concentrations in the subsequent experiments. Furthermore, it was observed that 3-DSC exerted a stronger inhibitory effect on 22RV1 cells than DU145 cells at equivalent concentrations.

To evaluate the effect of 3-DSC on the proliferation of two PCa cell lines over time, we performed a CCK-8 assay to determine cell proliferation following treatment with 3-DSC at varying doses (0, 10, 20, and 40 μM) for 24, 48, 72, and 96 h in 22RV1 and DU145 cells. The concentrations of 3-DSC were selected based on previously determined IC_50_ values. The results (Figures 8B,C) indicated significant suppression of cell growth in the 3-DSC treatment groups compared to the control group at 24, 48, and 72 h, with statistical significance observed (*p* < 0.05). Additionally, as the treatment dosage was increased, the proliferative inhibition of PCa cells by 3-DSC became more pronounced.

#### 3.7.2 3-DSC retrained migration of 22RV1 and DU145

Wound healing tests were used to examine PCa cell migration after the 3-DSC intervention. The findings revealed that different concentrations of 3-DSC effectively suppressed the migration ability of PCa cells after 24 and 48 h of treatment, with a stronger inhibitory effect observed at higher concentrations of 3-DSC (Figures 8D,E).

#### 3.7.3 3-DSC promoted apoptosis of 22RV1 and DU145

**FIGURE 9:**
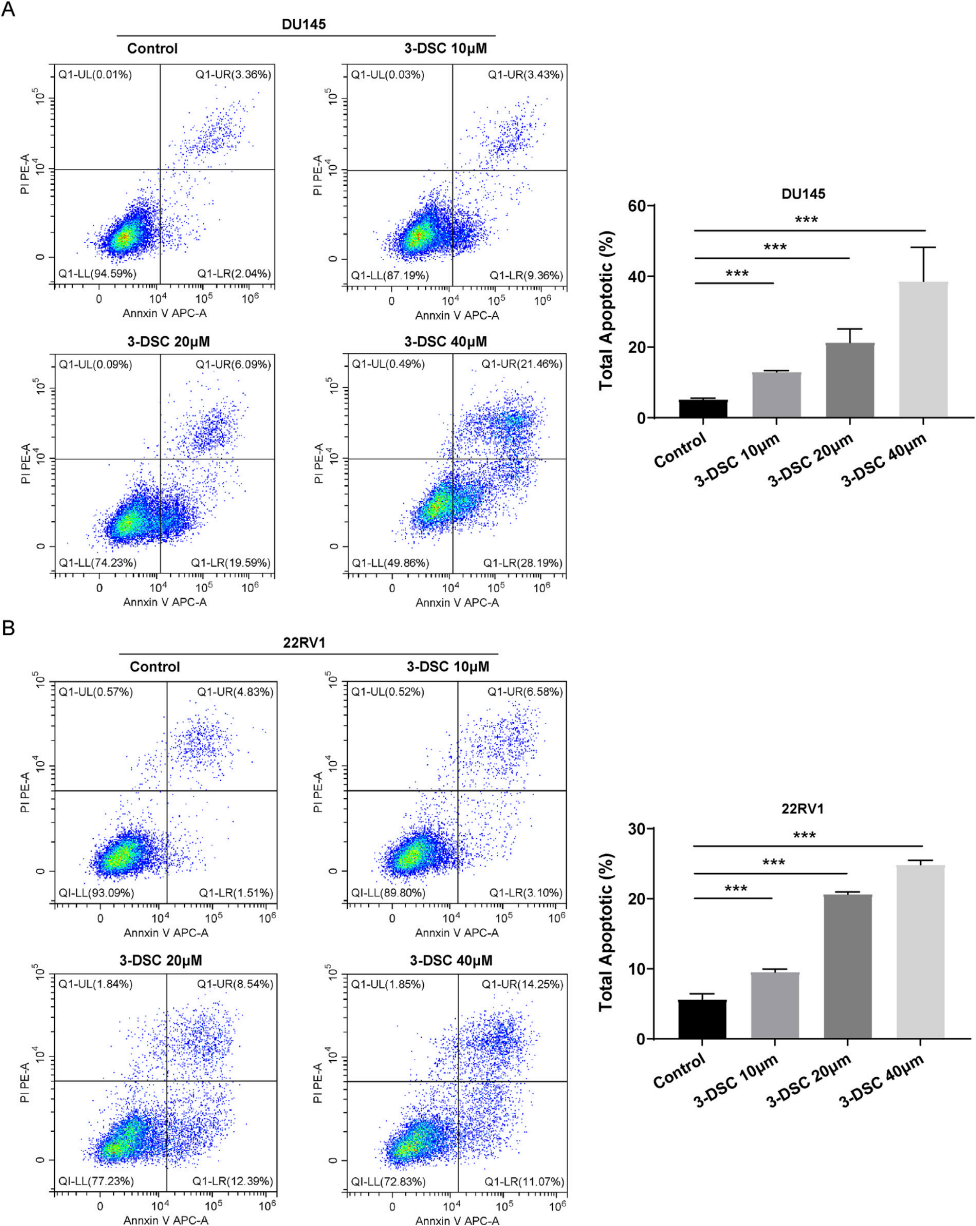
3-DSC promoted 22RV1 and DU145 apoptosis. Cells were treated with 3-DSC at concentrations of 10, 20, or 40 μM, and the total cell apoptosis detection was analyzed using flow cytometry. **(A)** Illustrations of DU145 undergoing apoptosis, accompanied by a quantitative analysis of the proportion of apoptotic cells in each group. **(B)** Illustrations of 22RV1 undergoing apoptosis along with a quantitative analysis of the proportion of apoptotic cells in each group. Compared to the control group, ***p < 0.001.

To investigate the potential correlation between the suppressive effect of 3-DSC on proliferative viability and apoptosis, flow cytometry was utilized to determine apoptosis of DU145 and 22RV1 cells (Figure 9). The total number of apoptotic cells significantly increased in the three concentration groups of 3-DSC compared to the control group, indicating the ability of 3-DSC to induce apoptosis in PCa. Additionally, the effect of facilitating apoptosis became more significant as 3-DSC concentration increased.

#### 3.7.4 3-DSC arrested DU145 and 22RV1 cells in the G2/M phase

**FIGURE 10:**
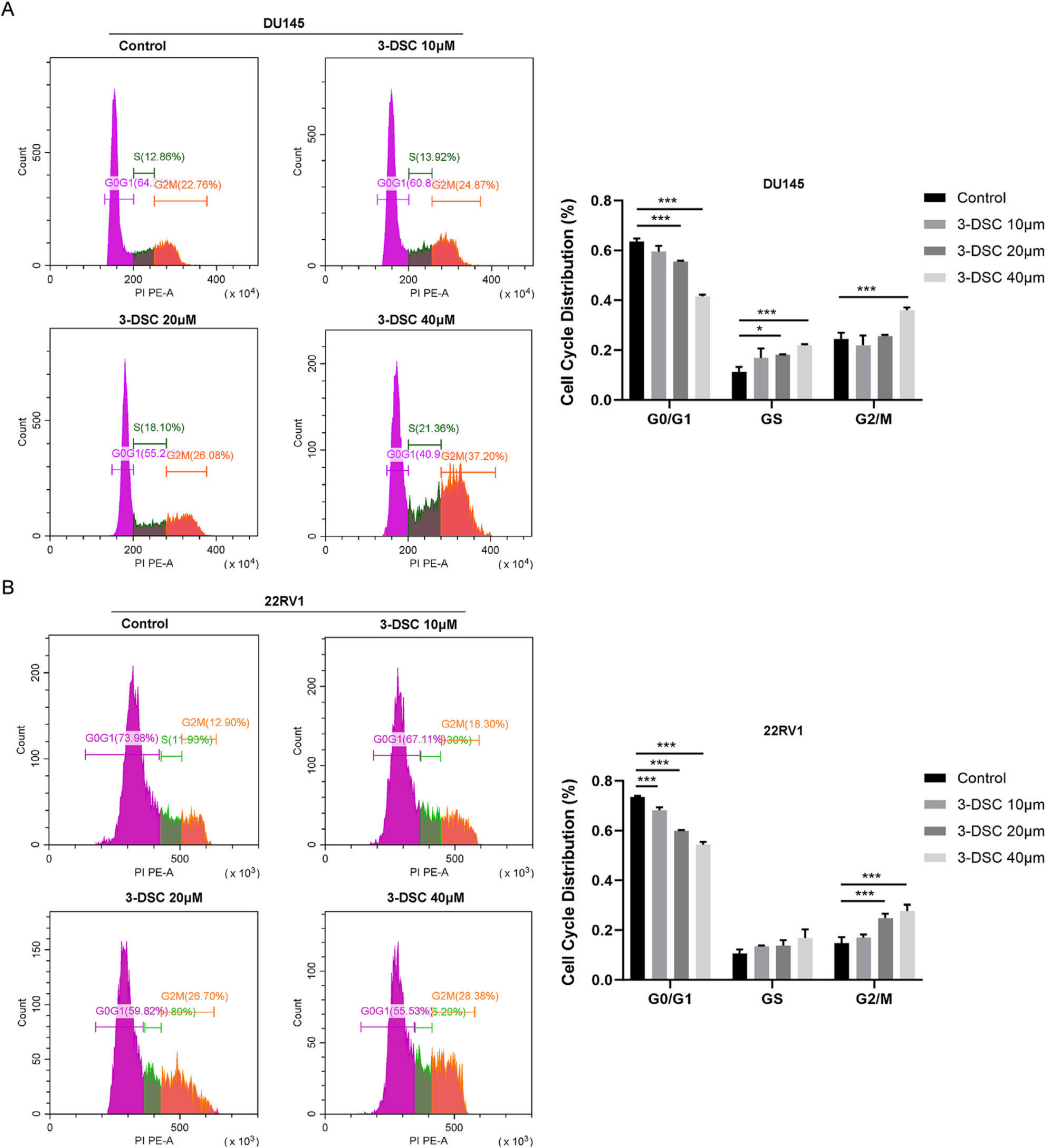
The treatment of 3-DSC resulted in S and G2/M phase cell cycle arrest in DU145 and G2/M phase cell cycle arrest in 22RV1. **(A)** Distribution of the typical cell cycle of DU145, with quantitative analysis of the proportions of cell cycle phases. **(B)** Distribution of the typical cell cycle of 22RV1, with quantitative analysis of the proportions of cell cycle phases. Compared to the control group, *p < 0.05, ***p < 0.001.

Furthermore, we evaluated the impact of 3-DSC on the cell cycle of PCa cells using flow cytometry experiments (Figure 10). Treatment of DU145 cells with 3-DSC resulted in cell cycle arrest during the S and G2/M phases, whereas 22RV1 cells were arrested during the G2/M phase. In the 3-DSC-treated group, there was a decrease in the proportion of DU145 cells in the G0/G1 phase and an increase in the proportion of cells in the S and G2/M phases compared to the control group. Similarly, 22RV1 cells exhibited a reduction in the proportion of cells in the G0/G1 phase and increased the number of cells in the G2/M phase in the group treated with 3-DSC. We concluded that 3-DSC primarily caused the PCa cell cycle to block at the G2/M phase, thus inhibiting cell proliferation.

#### 3.7.5 3-DSC regulated the expression of p53 pathway associated proteins

**FIGURE 11:**
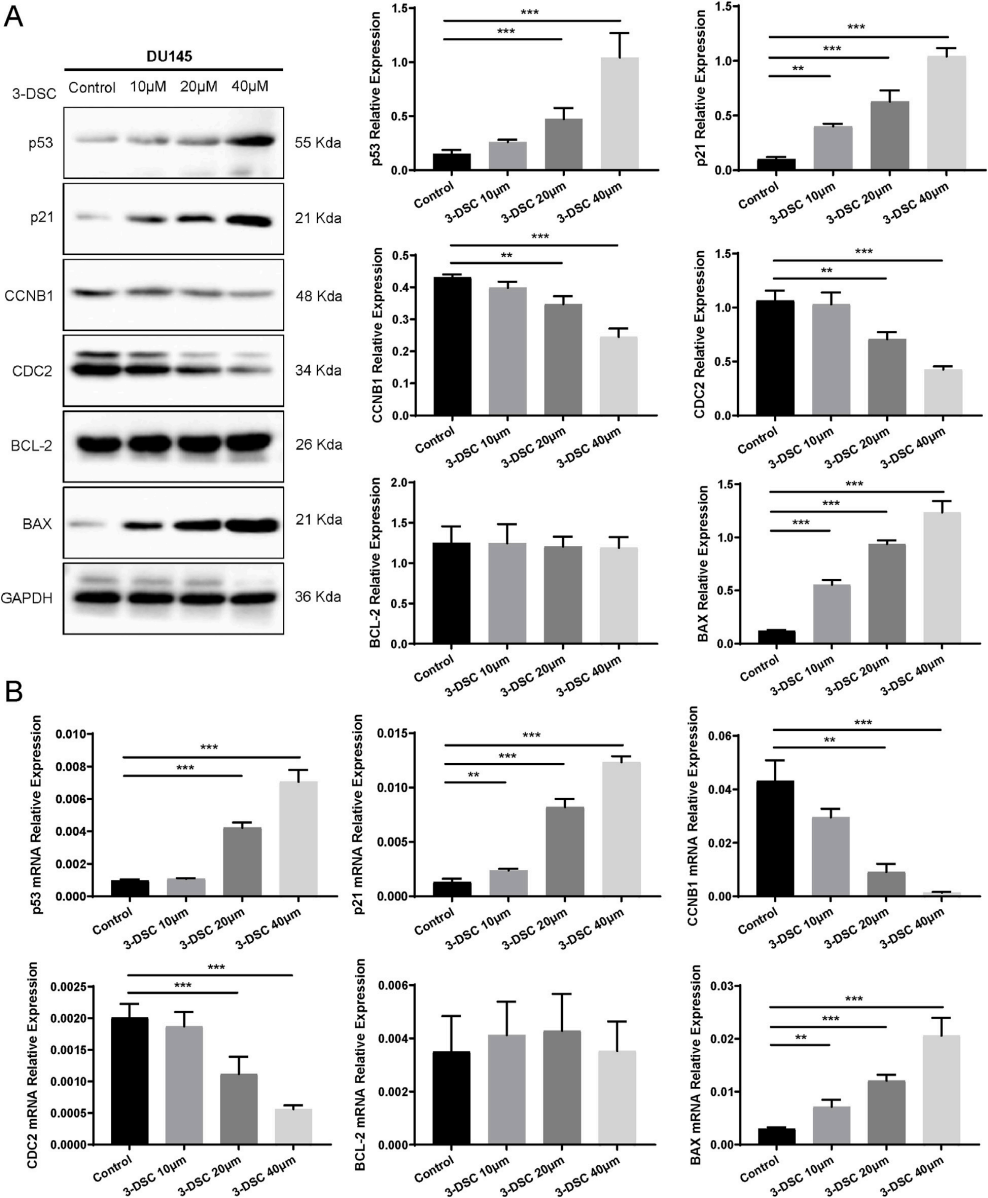
3-DSC downregulated CCNB1 and CDC2 and upregulated p53, p21, and BAX in DU145 cells. **(A)** Detection of p21, p53, BCL-2, BAX, CCNB1, and CDC2 protein expression levels of each group in DU145 cells via Western blotting. **(B)** Quantitative qRT-PCR of p21, p53, BCL-2, BAX, CCNB1, and CDC2 mRNA levels in DU145 cells. Compared with the control group, **p < 0.01, ***p < 0.001.

**FIGURE 12:**
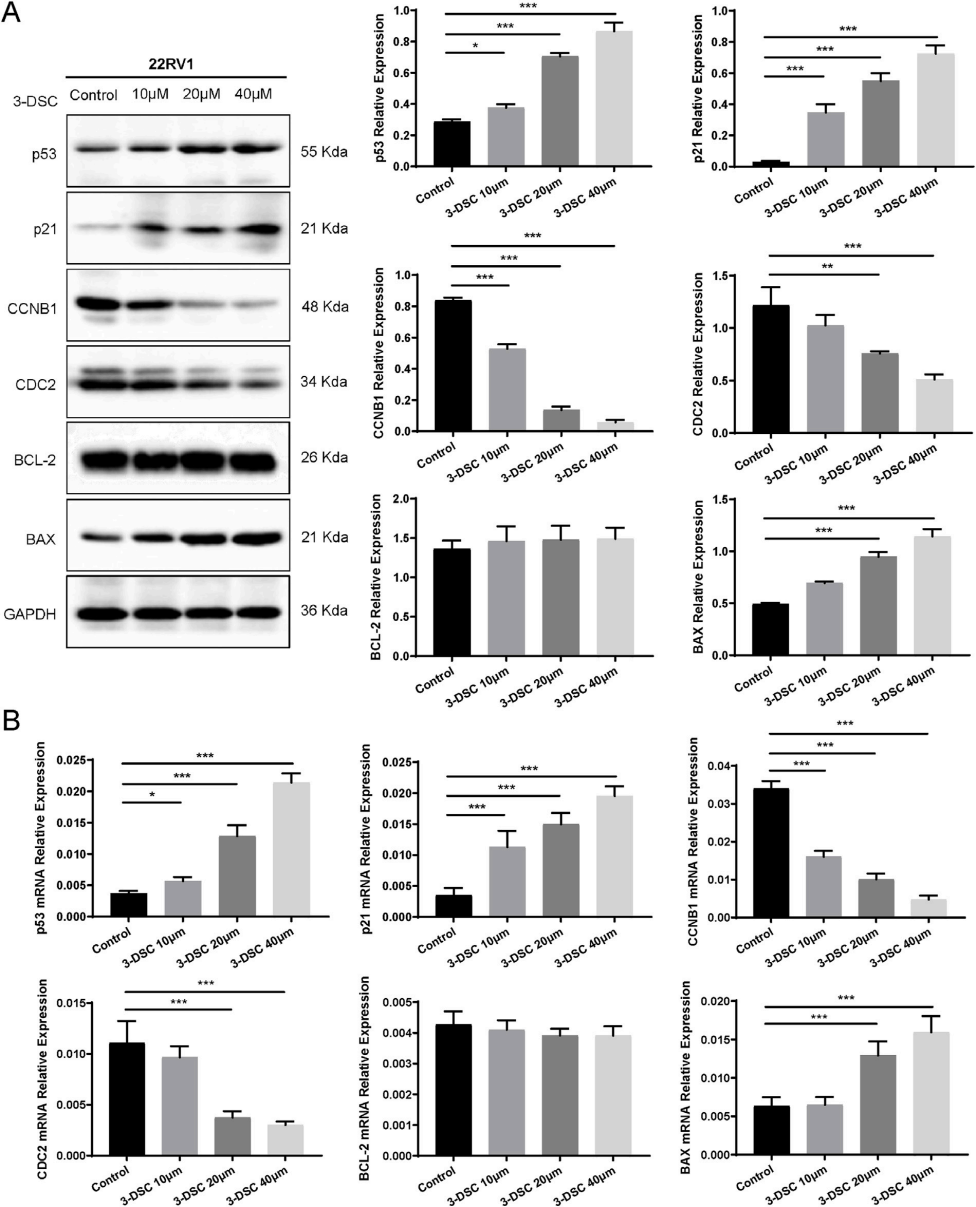
3-DSC downregulated CCNB1 and CDC2 and upregulated p53, p21, and BAX in 22RV1 cells. **(A)** Detection of p21, p53, BCL-2, BAX, CCNB1, and CDC2 protein expression levels of each group in 22RV1 cells via Western blotting. **(B)** Quantitative qRT-PCR of p21, p53, BCL-2, BAX, CCNB1, and CDC2 mRNA levels in 22RV1 cells. Compared with the control group, *p < 0.05, **p < 0.01, ***p < 0.001.

To further verify the role of 3-DSC in apoptosis promotion and proliferation suppression, cells were exposed to various doses of 3-DSC (10, 20, and 40 μM) for 24 h. The expression of proteins relevant to the p53 pathway was then examined using Western blotting. The results revealed that 3-DSC decreased CCNB1 and CDC2 protein expression and promoted p53, p21, and BAX expression in DU145 and 22RV1 cells (Figures 11A, 12A). Furthermore, the relative expression of CCNB1 and CDC2 proteins was negatively correlated with the concentration of 3-DSC. Conversely, p53, p21, and BAX proteins positively correlated with the concentration of 3-DSC. Additionally, 3-DSC did not impact BCL-2 proteins in DU145 or 22RV1 cells. We concluded that 3-DSC enhanced BAX expression and restrained protein expression of CCNB1 and CDC2 in PCa cells through the p53/p21 pathway, thereby regulating the cell cycle.

#### 3.7.6 3-DSC regulated the expression of p53 pathway associated mRNAs

We further validated p53 pathway-related mRNAs by qRT-PCR. The findings implicated that 3-DSC significantly reduced the levels of CCNB1 and CDC2 mRNAs and promoted p53, p21, and BAX mRNA expression in DU145 and 22RV1 cells. However, no effect was observed on BCL-2 mRNA. These findings are consistent with the alterations in protein expression observed (Figures 11B, 12B).

## 4 Discussion

PCa is the most prevalent non-skin malignancy in men worldwide, categorized into androgen-dependent and androgen-independent neoplasms. Patients often exhibit no obvious symptoms in the early stages of PCa, resulting in diagnosis at the advanced stages, where they are more susceptible to drug resistance and ultimately progress to fatal androgen-independent PCa. Therefore, novel therapeutic strategies for PCa that are less toxic and more effective are needed (Zhi et al., 2018).

Plants are the primary source of multiple chemotherapy drugs with potent antitumor properties (Ramzan et al., 2022). Combining plant-derived chemicals with conventional therapies exhibits promise in treating PCa (Mirzaei et al., 2022). Natural compounds have been an important source in the exploration of novel drugs for tumors. Recently, herb and botanical drug extracts have emerged as promising clinical adjuvant treatments for PCa, demonstrating advantages in reducing toxic side effects, lowering tumor recurrence rates, and enhancing patient survival quality (Wang et al., 2018). However, the precise molecular mechanisms underlying these benefits remain unclear.

*Sappan lignum*, the heartwood of Caesalpinia sappan L, is predominantly distributed in Southeast Asia**.** The chemical composition of *Sappan lignum* is known to be complex (Yang et al., 2016). In this study, 21 active ingredients of *Sappan lignum* were screened based on Lipinski’s rule of 5 and GI absorption. Compounds meeting Lipinski’s rule of 5 for oral drug-likeness and GI absorption possessed improved bioavailability and pharmacokinetic characteristics. However, if the compounds reach high concentrations, they can be efficiently absorbed and exert therapeutic effects (Ye et al., 2022). Therefore, it is necessary for us to conduct serum chemistry analysis on *Sappan lignum* in future research (Ye et al., 2023). The phytochemical compounds that complied with the requirements primarily consisted of flavonoids, alkaloids, saponins, terpenoids, and additional components, including diphenyls, steroids, and fatty acids. Our investigation revealed that the target proteins for *Sappan lignum* primarily included kinase (25.34%) and enzyme (17.64%).

*Sappan lignum* has diverse biological actions, including anti-inflammatory, antitumor, immunosuppressive, and antioxidant properties (Jung et al., 2015; Yang et al., 2019). Extracts of *Sappan lignum* exhibit apparent inhibitory effects on various tumor cells and possess diverse applications. Recent studies have demonstrated that Caesalpinia sappan L. major extract increases the generation of reactive oxygen species (ROS) and releases apoptosis-inducing factors in human colon cancer cells HCT116, suppressing proliferation and causing apoptosis (Seo et al., 2020). The ethyl acetate extracts of *Sappan lignum* demonstrated the ability to induce cell cycle arrest at the G2/M phases and cell mitosis in lung, colon, and liver cancer cells *in vitro* while also inhibiting tumor necrosis factor-alpha/nuclear factor kappa-B cellular signaling to exert antitumor effects (Zhang et al., 2014). Moreover, neck cancer cell viability was significantly reduced in response to the chloroform extract of *Sappan lignum.* This antitumor effect is consistent with our findings and is ascribed to activating the apoptosis-related proteins p53 pathway, which induces apoptosis in tumor cells (Kim et al., 2005). 3-DSC is one of the main active ingredients of *Sappan lignum* with the molecular formula of C_16_H_16_O_5_. The strongest binding affinity of 3-DSC to the primary targets of PCa was determined by molecular docking, indicating its potential as an active small molecule for treating PCa. 3-DSC has been confirmed to inhibit proinflammatory and anti-allergy activities. It promoted the arrest of the G2/M cell cycle, cell apoptosis, generation of ROS, and increased expression of cell cycle regulators (Kwak et al., 2021). Studies also have implied that 3-DSC increased mitochondria-related intrinsic apoptosis to restrain skin tumor cell growth (Fu et al., 2021). Besides, 3-DSC caused cell cycle arrest in colon and lung cancer cells by reducing the expression of CDC2, p27, and CCNB1 (Zhao et al., 2019; Lee et al., 2023).

Through intersecting the targets of *Sappan lignum* with 821 DEGs obtained from the public clinical datasets of PCa, eight core targets of *Sappan lignum* against PCa were retrieved, containing BCL-2, PTGS2, CCNB1, PGR, AURKA, MET, NR3C1, and ABCG2. The KEGG enrichment analysis indicated that tumor-associated p53 was the primary treatment pathway, including BCL-2 and CCNB1. p53 is an important tumor suppressor protein that regulates tumor cell cycle arrest, apoptosis, and DNA repair (Balakrishnan et al., 2006). p53 activation can increase the expression of its downstream effector molecules, p21 and BAX. p21 is a cell cycle inhibitor that prevents cell cycle progression by inhibiting the activity of the cyclin-cell division cycle gene complex (Jin et al., 2020). BAX is a pro-apoptotic protein that promotes apoptosis by interacting with the anti-apoptotic protein BCL-2 (Zhao et al., 2022).

In the *in vitro* part, we found that 3-DSC resulted in the proliferation and migration inhibition and apoptosis promotion of PCa cells, and the antiproliferative impact on PCa cells was enhanced with increasing concentrations of 3-DSC. Besides, we observed that 3-DSC promoted p53 expression in PCa cells, indicating that the p53 pathway was activated. p21, a downstream target gene of p53, was also significantly upregulated, which further decreased the expression of CCNB1-CDC2, leading to G2/M phase cell cycle arrest, thereby inhibiting tumor cell proliferation (Uzor et al., 2021; Chen et al., 2022)**.** Dysregulation of CCNB1 and CDC2 is related to tumor cell cycle arrest, a process governed by the upstream protein p53 (Long et al., 2016). Additionally, this study showed that 3-DSC dramatically raised BAX levels in PCa cells, both in terms of protein and mRNA. The BCL-2/BAX ratio is crucial in the apoptosis regulation (Zhang et al., 2010). The results of KEGG pathway analysis and experimental data were consistent, indicating that 3-DSC exerted anti-proliferative and pro-apoptotic effects in PCa cells by through the p53/p21/CCNB1/CDC2 signaling pathway. These results highlighted the potential therapeutic benefits of *Sappan lignum* in PCa treatment.

This study selected human PCa cells DU145 and 22RV1. 22RV1 cells belong to androgen-independent PCa cell lines that are suppressed by reduced androgens (Duan et al., 2015), while DU145 cells are androgen-unresponsive and exhibit androgen-independent growth, indicating a poorer prognosis (Zhang et al., 2019). The 22RV1 and DU145 cell lines can validate the potential therapeutic effects of drugs against prostate cancer and are valuable for modeling different types of PCa (Cheng et al., 2021). In the network pharmacology analysis, 32 targets of *Sappan lignum* against prostate cancer were identified, including SRD5A2, which is closely related to the androgen receptor (AR). In the PCa

Patients, the expression level of SRD5A2 is often elevated, leading to increased dihydrotestosterone (DHT) levels, thereby enhancing AR activity and affecting cell cycle and proliferation (Li et al., 2011). Combining the results of KEGG enrichment, we believe that *Sappan lignum* may regulate AR activity and function through SRD5A2, thereby co-regulating the cell cycle with the p53 pathway, ultimately inhibiting proliferation of androgen-independent PCa cell. Quercetin, another flavonoid of *Sappan lignum*, was able to inhibit AR protein expression in androgen-responsive prostate cancer cell lines, thereby suppressing the expression of prostate-specific, androgen-regulated tumor markers (Xing et al., 2001). This inhibition leads to reduced tumor proliferation through cell cycle regulation (Yang et al., 2015). The specific mechanisms of *Sappan lignum* need further *in vitro* experiments to verify AR-related gene regulation.

HPA and TCGA clinical data indicated elevated levels of CCNB1 in patients with PCa. Gene silencing of CCNB1 suppresses proliferation and promotes apoptosis by activating the p53 signaling pathway in tumor cells (Zhang et al., 2018). The expression level of CCNB1 varied among 501 PCa patients with different TMN stages and Gleason scores, with significantly higher upregulation observed in patients with T3 stage, N0 stage, and Gleason score of 7. Studies have reported that CCNB1 is a potential prognostic marker for recurrence following radical prostatectomy (Ersvær et al., 2020). These results confirmed that the inhibitory potential of 3-DSC for PCa provided a clinical advantage and suggested a potential new application of 3-DSC as an antitumor drug, with important implications for developing novel anticancer drugs.

There are some limitations in this study. Network pharmacology, bioinformatics, and *in vitro* experiments consistently verified and explored the main capacity of 3-DSC for proliferation suppression. However, in-depth investigation is required to comprehensively understand alternative plausible mechanisms of action. In subsequent investigations, a PCa xenograft mouse model

Should be employed to further examine the precise effects of 3-DSC, with an increased focus on *in vivo* studies. To further explore additional signaling pathways and targets influenced by *Sappan lignum*, multi-omics technologies can be utilized, having a clear understanding of its anti-cancer mechanism.

## 5 Conclusion

This study identified the active compound in *Sappan lignum* with anti-PCa properties. We found that the core compounds, specifically 3-DSC, could exert a therapeutic effect on PCa through the p53 pathway. *In vitro* experiments confirmed that 3-DSC exhibited significant antiproliferative, pro-apoptotic, and G2/M phase cell cycle arrest effects in 22RV1 and DU145 cell lines, potentially through modulating the p53/p21/CDC2/CCNB1 pathway. Additionally, clinical data analysis indicated that CCNB1 is closely associated with the clinicopathological characteristics of PCa patients, further supporting the scientific basis for 3-DSC as a potential therapeutic agent. Our multi-level validation study provides crucial theoretical support and experimental basis for a new application of *Sappan lignum* as an antitumor drug.

## Data Availability

The original contributions presented in the study are included in the article/supplementary material, further inquiries can be directed to the corresponding authors.
